# The Effect of Bread Fortification with Whole Green Banana Flour on Its Physicochemical, Nutritional and In Vitro Digestibility

**DOI:** 10.3390/foods9020152

**Published:** 2020-02-05

**Authors:** Amir Amini Khoozani, Biniam Kebede, John Birch, Alaa El-Din Ahmed Bekhit

**Affiliations:** Department of Food Science, University of Otago, 9016 Dunedin, New Zealand; biniam.kebede@otago.ac.nz (B.K.);

**Keywords:** green banana, flour, bread, digestibility, nutritional, mineral, resistant starch

## Abstract

The use of Whole Green Banana Flour (WGBF) in bread production may be a strategy to improve the nutritional profile of bread, but the extent of improvement may depend on the processing conditions of the flour. Therefore, WGBF was produced using two methods (freeze-drying and air-oven drying) and was used in bread-making. This study investigated the effect of flour type—FDF (WGBF produced by freeze-drying) and ODF (prepared by air-oven drying at 50 °C)—at fortification levels of 0% (control), 10%, 20%, and 30% on the fortified bread. A significant decrease in energy caloric value and an increase in moisture and fibre at >20% fortification level (*p* < 0.05) was noted. The ODF bread samples had a higher browning index compared to the control and the FDF samples. Addition of WGBF improved macro minerals (Mg, Ca, Na, K, and P) with a no significant change in micro minerals (Fe, Zn, and Mn). The use of FDF in bread resulted in a marked increase in resistant and slow digestible starch levels in F30 compared to ODF samples and their comparable fortification levels. The digestibility of the bread samples showed that WGBF can be used as an alternative functional ingredient to prepare bread with better nutritional value.

## 1. Introduction

Banana is one of the most favourite fruits in the world and has a high nutritional value. Banana contains higher flavonoids, dietary fibre (DF), and resistant starch (RS) at its early stage of ripeness than when its ripe [1]. One of the important features of RS is that of not being digested within 2 h in the intestine and it enhances the growth of probiotics in the large intestine, which may have a positive indirect effect on colorectal cancer [2]. A whole green banana including the peel is considered as a rich source of RS (41%–59%) [3]. Considering the undesirable taste of green banana, green banana flour production has been recommended to add value to banana crops in terms of reducing waste and loss during the production chain, improve sustainability, and capture some of its nutritional benefits that are lost on ripening [4]. Recently, it has been reported that the type of heating process used in the whole green banana flour preparation has an impact on both the physicochemical and technological properties of the flour [5]. For instance, the RS content in banana flour can vary from 20% to 59% based on the banana species, stage of ripeness, and most importantly, the drying treatment applied [6,7].

In order to deliver additional health benefits using this functional ingredient, a mixture of banana flour and other alternative flours has been proposed in mostly starch-based food products, such as pasta [8], bread [9,10,11], cake [12,13,14], and gluten-free products [15,16,17].

Bread is extensively consumed all over the world as it contributes to the intake of proteins, lipid, and carbohydrates especially in developing countries. Thus, there is a good opportunity to use banana flour to fortify bread and to increase its nutritional value [18].

Even though a wide range of bread types are available on the market, white bread is still the first choice of many consumers due to its sensory attributes. However, white bread is considered as a high glycaemic index food due to its high percentage of rapidly digested starch (RDS), which is positively correlated with the in vivo postprandial glycaemic index (pGI) after 20 min of digestion [19]. On the other hand, slow digestible starch (SDS) accounts for starch digested between 20 to 120 min according to the definition provided by Englyst, et al. [20]. As recent studies have shown notably higher content of SDS and RS compared to RDS in green banana pulp flour, utilization of it in bread has gained attention as of late [11,21,22,23]. The slow rate of SDS and RS digestion can improve the insulin response which controls metabolic syndrome and contributes to the risk reduction of obesity, diabetes, and cardio-related diseases [24].

Although there are few reports on the effect of replacement of wheat flour with green banana flour on bread’s technological properties [25], in vitro studies investigating the digestibility of starch in this model are scarce. Furthermore, the available information on the effect of the green banana flour preparation method on bread properties is deficient. In order to maximize the yield of green banana flour and take advantage of its high nutritional value, the use of both peel and pulp are proposed [26]. The objectives of this study were to determine the effect of both the heat treatment used in drying and the fortification levels of whole green banana flour (WGBF) on the physicochemical and nutritional properties as well as the digestibility of bread containing WGBF.

## 2. Materials and Methods

Banana, wheat flour, and all the ingredients required for bread making (baking powder, commercial dry yeast, butter, salt, milk, and honey) were purchased from a local store (Dunedin, New Zealand).

### 2.1. Banana Flour Production

Green banana flour was produced from whole green banana (*Cavendish of Musa AAA group*), including its peel, by two drying methods, freeze-drying (FDF) and air-oven drying at 50 °C (ODF), as reported our previous study [7]. The proximate composition of the flour samples (g/100 g db) was as follows: FDF (protein 3.97, lipid 0.92, ash 5.21, carbohydrates 84.61, moisture: 5.27); ODF (protein 4.17, lipid 0.93, ash 5.19, carbohydrate: 84.62, moisture: 5.09); wheat flour (WF) (protein 9.78, lipid 1.24, ash 2.83, carbohydrate: 76.68, moisture: 9.47). All flour samples were screened using 250 μm sieves, vacuumed packed in polypropylene packs and stored in a refrigerator for the bread-making process.

### 2.2. Bread Making

Bread preparation was conducted using a protocol suggested by Ghorbel, et al. [27]. All the ingredients were equilibrated at ambient temperature for 20 min before starting the process. Wheat flour was mixed with FDF or ODF at replacement levels of 10%, 20%, and 30% (w/w). A control bread group was prepared using 100% wheat flour.

All the dry ingredients were mixed for 3 min at a low speed in a bread maker (Breville, Sydney, Australia) followed by the addition of milk, butter, and water and mixing for further 12 min at medium speed. The bread samples were prepared as follows for making 100 g control dough sample: 53.2 g of WF, 16 mL of water, 16 mL of milk, 0.5 g of sodium chloride, 5 g of honey, 8.8 g of butter, and 0.5 g of activated dried yeast. Treatments were made with 10%, 20%, and 30% substitution of WF with WGBF. After kneading, the dough was proofed for 90 min at 37 °C. Baking was completed after 25 min of heating at 180 °C followed by cooling at room temperature for 1 h.

### 2.3. Bread Physicochemical Properties

All the physicochemical properties of bread samples were determined using the approved AOAC 2000 standard methods [28]. The moisture content was assayed using a vacuum oven dryer (Thermoline, Australian Marketing Group, Marrickville, NSW, Australia) at 60 °C for 16 h. The water activity was measured by an Aqualab device (Decagon Devices, Washington DC, USA) at 25 °C (Method 925.40; AOAC 2000). Ash content was measured using a furnace according to method 923.03; AOAC 2000. Total fat content was measured using the Soxhlet extraction method (Method 920.39; AOAC 2000). Protein content was conducted using the Kjeldahl method to determine total nitrogen and a conversion factor of 6.25 was used (Method 923.03; AOAC 2000). The carbohydrate and energy values were calculated via the following formula:

Carbohydrate (g)=100−(moisture content  (g)+ash (g)+protein (g)+lipid (g))

Energy value (Kcal/100 g)=4×protein (%)+9×lipid (%)+4×carbohydrate (%)

Total dietary fibre, soluble dietary fibre, and insoluble dietary fibre contents (TDF, SDF, and IDF, respectively) were determined using Megazyme kit (K-TDFR; Megazyme International, Wicklow, Ireland) based on AOAC Method 991.43 and AACC Method 32-07.01. Briefly, 1 g of ground dried bread was subjected to heat stable α-amylase under 100 °C cooking with an incubation at 60 °C with Bacillus licheniformis protease and amyloglucosidase afterwards. After depolymerisation of proteins and hydrolysis of starch, insoluble dietary fibre was filtered and washed with distilled warm water. The residue was dried and weighed. A hydrolysed sample was precipitated with ethanol, filtered, washed with ethanol, and dried for measuring SDF. The total dietary fibre value was corrected for protein and ash contents.

### 2.4. Bread Crust and Crumb Colour

The colour of the breads’ crust and crumb was measured by a Hunterlab Spectrocolorimeter (Hunter Lab MiniScan Plus Colorimetric, Virginia, WV, USA). Colour was measured at three points of the fresh bread samples in triplicates. The results were determined using the CIE L* a* b* system, where L* (from pitch black, 0, to perfect white, 100), a* (from –a* greenness to +a* redness), and b* (from –b* blueness to +b* yellowness) parameters. Chroma, browning index and ∆E were calculated by the following formulas [29].

$$\mathrm{Chroma}=\sqrt{{a*}^{\;2}{+b*}^{\;2}}$$

$$\mathrm{Browning}\;\mathrm{index}=\frac{100\;(x-0.31)}{0.17}$$

where x was obtained by the following formula:

$$x=\frac{a^{*\;}+{1.75\;L}^{*}}{{5.645\;L}^{*}{+a}^{*}-{3.012\;b}^{*}}$$

$$\text{∆}E=\sqrt{{(\Delta L*)}^{2}+(\Delta {a*)}^{2}+{\left(\Delta b*\right)}^{2}}$$

### 2.5. Mineral Profile

The analysis of minerals was performed by an Agilent 7500ce quadrupole inductively coupled plasma mass spectrometry (ICP-MS) mass spectrometer connected to a CEM MARS6 programmable 2450 MHz microwave reaction system with a selectable operator output of 0–1600 W (CEM Corporation, Matthews, NC, USA). Approximately 2 mg of dried bred samples were weighed in a MARSX press (CEM Corporation, Matthews, NC, USA) digestion tube with the addition of 10 mL of concentrated quartz distilled HNO_3_ subsequently. After digestion of the samples in a microwave, they were spiked offline with a cocktail of 7 reference elements to compensate for any drift or possible matrix effects. Serial dilutions of a SPEX CertiPrep multi-element were prepared for calibration standards (Spex Certiprep, Metuchen, NJ, USA). The ICP-MS was tuned according to the manufacturer’s recommendations for minimizing the instrumental interferences. The accuracy of the measurement was established with a Certified Reference Material (bovine muscle powder, reference material 8414, Agriculture Canada, Distributed by The National Institute of Standards and Technology) following the method of Anyasi, et al. [30].

### 2.6. In Vitro Starch Digestibility Tests

Based on its digestion, starch has been classified into rapidly digestible starch (RDS, amount of released glucose after 20 min); slowly digestible starch (SDS, amount of released glucose between 20 and 120 min), and resistant starch (RS, starch remaining undigested after 180 min) [31]. All starch determinations were analysed using Megazyme kits (Megazyme International, Wicklow, Ireland) based on the official method AOAC modified by Sharma, et al. [32]. The released glucose was measured at 0, 0.5, 1, 1.5, 2, and 3 h of digestion to imitate starch enzymatic hydrolysis in mouth, stomach, and small intestine. Firstly, 100 mg bread samples were subjected to 0.2 mL artificial porcine salivary amylase solution (1 g/10 mL of buffer KCl–HCl solution in 0.02 M phosphate buffer at pH 1.5.) Then incubation of the samples was carried out at 37 °C for 30 min after the addition of pepsin (1 mg/mL) enzyme prepared in 0.02 M HCl (40 mg α-amylase A-3176; Sigma, Madrid, Spain) to mimic the gastric phase. Lastly, in order to mimic the intestinal phase, samples were incubated at 37 °C for 2 h in the presence of pancreatin (enzyme/starch ratio, 1.3:100, *w*/*w*) and amyloglucosidase (enzyme/starch ratio, 0.26:1, v/w) prepared in 0.2 M sodium acetate buffer (pH = 6) were added and the released glucose was measured using glucose oxidase–peroxidase reagent (GOPOD). [33]:

$$\mathrm{Digestible}\;\mathrm{starch}\;(g/100\;g\;\mathrm{dwb})=\frac{0.9\times \mathrm{glucose}\;\mathrm{concentration}\times \mathrm{volume}\;\mathrm{of}\;\mathrm{digesta}\;(\mathrm{mL})}{\mathrm{Sample}\;\mathrm{weight}\;\left(g\right)\times \mathrm{starch}\;(g)}$$

#### 2.6.1. Total Starch Analysis of Bread

An amount of 100 mg of dried ground bread samples was mixed with 2 M KOH at 25 °C for half an hour. After the addition of 0.4 M sodium acetate buffer pH 4.75 and 60 mL amyloglucosidase (AMG), the suspension was incubated for 45 min at 60 °C in a shaking water bath. The total starch content was measured as the released glucose with glucose oxidase peroxidase (GODPOD) assay and was determined at 510 nm.

#### 2.6.2. Resistant Starch Content of Bread

In brief, 100 mg of bread samples was incubated with maleate buffer (50 mM, pH 6) and pancreatic α-amylase/AMG at 37 °C for 4 h. After stopping the starch hydrolysis reaction by adding ethanol and centrifugation, the pellet was dissolved in 4 M KOH by vigorous stirring in an ice-water bath over a magnetic stirrer. Digested pellet and supernatant were incubated separately with AMG at 50 °C for 20 min in a water bath. Finally, the absorbance of the released glucose was measured by GODPOD assay at 510 nm against the reagent blank. Resistant starch content was calculated as mg of glucose × 0.9 [21].

### 2.7. Statistical Analysis

Data were analysed using ANOVA to determine the effects of WGBF type, the addition percentage on the measured parameters. The statistical analysis was performed by Minitab^®^ program version 16. Post-hoc Tukey test was used to determine the differences among the means at 95% of confidence level. The results are the means of three replicate experiments and expressed as mean and standard error of mean (SEM).

## 3. Results and Discussion

The results shown in Table 1 present the proximate chemical composition of bread samples. The fortification above 10% caused a significant increase in water activity (a_w_) of the bread samples compared to the control ones. The moisture content (MC) and TDF values increased (*p* < 0.05) with the increasing WGBF fortification level in bread. This, suggest the presence of more hydrophilic chains that resulted in higher water absorption capacities [34]. These findings were in agreement with the results obtained by Eshak [11] and Gomes et al. [9], who used hydrocolloids as water binders. Protein and fat contents decreased with the increased addition of WGBF regardless of its preparation method (*p* < 0.05). This was expected since the protein content was lower in the WGBF (approximately 36% less in F30 and O30) compared to the WF [7]. This agrees with the findings reported by Gomes et al. [9] who utilised WGBF and Loong and Wong [35] who used green banana pulp flour.

High ash content in banana bread samples is indicative of the presence of high mineral components. The higher ash content in WGBF compared to WF improved the ash content in bread and resulted in a two and half times increase in the ash content for F30 and O30 samples (Table 1). From the results obtained from TDF and EV, it can be seen that higher content of TDF gave rise to less EV of the final product. Bread samples made with FDF displayed higher TDF compared to ones made with ODF in all fortification levels (*p* < 0.05). This result may be explained by the fact that fibres were preserved more during freeze-drying compared to air oven-drying [7]. Also, surface furrowing and cracking stems from heat treatment can be the cause of lower TDF in all ODF and eventually in the bread [36]. The findings of chemical composition indicated that WGBF-fortified bread samples had superior nutritional value than the control bread with a decrease in protein content.

### 3.1. Bread Colour Analysis

The control sample showed the L* value of 66.6 and 72.5 which indicates the whiteness of crust and crumb bread slice, respectively (Table 2). In relation to the browning index of bread crust, a significant increasing trend was observed in oven-dried fortified bread samples compared to freeze- dried ones. Although this trend was similar in the breads fortified with FDF as well, breads with freeze dried flour showed lower browning index, yellowness, and redness (Table 2). Previous findings have shown that chlorophyll was more preserved in FDF compared to ODF [7]. Crust colour saturation exhibited a declining trend with more WGBF content levels in bread. Martínez-Castaño, et al. [37] also reported the brownest colour in crust for gluten free bread made with partial banana flour substitution (15%). Control samples showed higher crumb yellowness and a more intense crust browning compared to all freeze-dried fortified bread samples (*p* < 0.05). The presence of different xanthophylls and phenolic compounds might be associated with the yellowish-brownish colour of the banana bread samples [10]. Also, Mohamed, et al. [38] mentioned that for fruits like banana that contain high levels of polyphenol, oxidase brings about enzymatic oxidation of mono-phenolic compounds and releases o-diphenols and o-quinones. The colour differences between control and fortified samples increased (*p* < 0.05) with increasing WGBF in bread. The higher carbohydrate content in WGBF also may have resulted in the Millard reaction specially in OD-bread crumb samples similar to that found in the literature [6,35,39].

### 3.2. Mineral Profile

**Figure 1:**
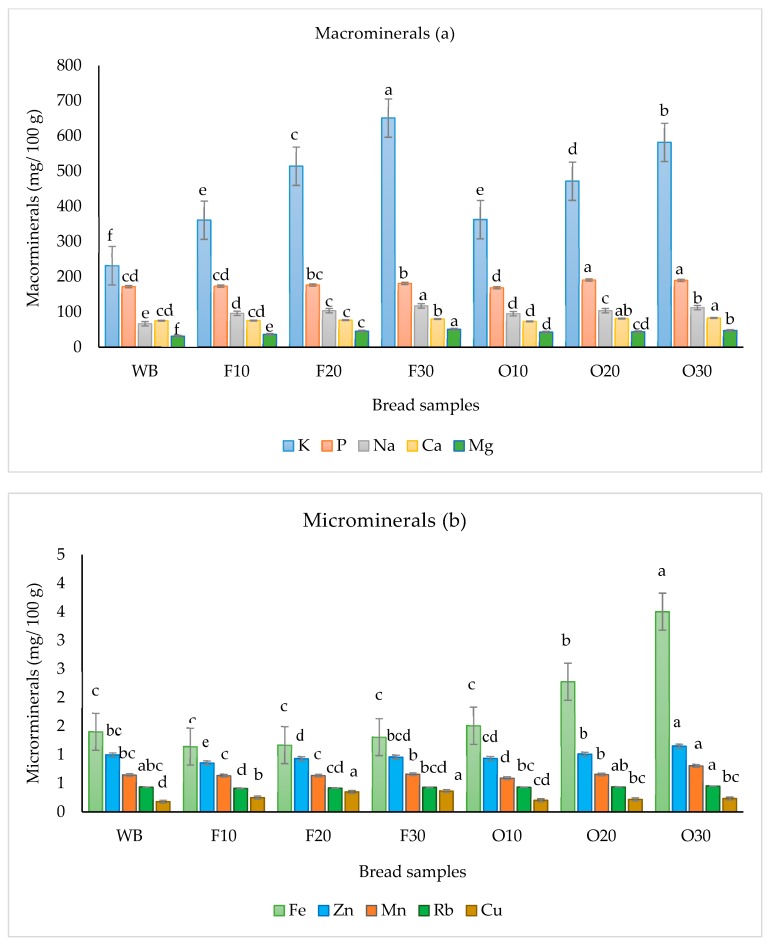
(**a**) Macro minerals in bread sample (**b**) Microminerals in bread samples. Bars for each mineral that have different letters are significantly different (*p* < 0.05).

Minerals play a key role in enzyme systems and facilitate the functioning of certain organs [40]. Some minerals are essential in larger amounts (macro) and others in smaller (micro) quantities. As more WGBF was incorporated into the bread, the mineral contents were increased, especially the macro elements (Figure 1). All the 20% and 30% bread samples contained significantly higher K, P, Na, Ca, and Mg compared to control (*p* < 0.05). Considering recommended daily intake of minerals [41], each 100 g of F30 sample accounts for 18.6% K, 18.16% P, 4.91% Na, 8.07% Ca, and 14.74% Mg. Also, all oven-dried fortified bread samples showed a higher percentage of Fe, Zn (O20 and O30), and Cu compared to control breads (Figure 1b). These statistically significant increases (*p* < 0.05) are due to the high ash content of WGBF [7,30]. These findings can be beneficial especially for products which are made for celiac disease [4]. The same observation was reported in bread made with fermented banana flour [42]. Correspondingly, Ho, et al. [43] reported an increment in Na, K, Mg, and Ca contents in bread made with 10% peeled yellow banana pseudo-steam flour in the presence of sodium carboxymethyl cellulose. Apart from these studies, there is no similar work in this area with which to compare the result. These results indicated that addition of WGBF, especially that produced by freeze-drying, could be utilized as a supplement to improve the essential minerals in bread products.

### 3.3. Starch Digestibility Analysis

It is well known that food products with high level of RS and SDS do not produce postprandial hyperglycemic and hyperinsulinemic spikes associated with RDS [44]. Control bread displayed low RS content (2.17%), which agrees with results from studies with breads [43,45]. As the amount of WGBF increased in bread, the RS% increased significantly, with the F30 samples (8.55%) having the highest percentage (*p* < 0.05). Lower RS content in O10, O20, and O30 samples was possibly due to the lesser amount in the ODF compared to the FDF [7]. Based on the recommended daily intake of RS for prebiotic effect, which is 6–12 g/d [2], having a 100 g bread of F30 and O30 can have this beneficial effect. The retrogradation process which occurs after cooling the bread samples and the formation of the amylo-lipid bonds creates RS type 3 and 5, respectively [37]. Furthermore, the presence of more DF in banana bread samples, specially above the 20% level, resulted in a change in the viscosity, which acts as a physical barrier on the starch surface and prevents it from being attacked by digestive enzymes [46]. As can be seen in Table 3, the amount of SDS increased significantly in O30 and F30 compared to other treatments. It has been mentioned that RS has a “sponge role” and is capable of absorbing water in the big intestine and helps in mixing with the foodstuff in a tangled network form, and thereby can slow down the rate of digestion [47].

Roman et al. [45] reported the remaining RS content cannot be beyond 2% w/w in bread made with 20% banana starch. Likewise, De Souza Viana et al. [18] reported 25% of substitution of WF with green banana pulp flour resulted in maximum 3.22% of RS in the final bread. In a study on an additional gluten-added bread made with 60% GBPF, a low RS was reported of 6.7%, which was explained by the low level of RS in the prepared flour (17%) [47]. In a recent work conducted by Agama-Acevedo et al. [24], bread crust was analysed for the remaining RS and it was found that no more than 2.5% was available. Similarly, Hernández-Aguirre et al. [4] reported 3.5% of RS in a gluten free bread but without reporting the RS content of GBPF.

## 4. Conclusions

It can be concluded that the use of whole green banana flour up to 30% in substitution for wheat flour significantly increased total dietary fibre, ash, and mineral content in bread. Additionally, bread samples made with air-over dried WGBF exhibited darker colour in both crumb and crust, with similar yellowness compared to the control bread sample in 10% level of fortification. The marked increase in macronutrients, P, K, Mg, Na, and Ca in particular, with a slight increase in micro minerals in all WGBF bread samples, specially above 10% fortification, showed an improvement in nutritional value of all fortified bread samples. The addition of 20% and 30% of WGBF in the bread resulted in a product with the pronounced percentage of RS and SDS. As FDF-bread samples showed lower RDS and DS compared to OD-bread samples, it might be concluded that the 30% WGBF addition can be considered as functional bread containing prebiotic dosage. Current findings are also expected to help the sustainability of food systems by the use of whole green banana and prevent losses during maturation, transportation, and storage. Also, previous studies have shown the synergic effect of hydrocolloids with resistant starch content which can be used for future studies on bread formulation.

## Figures and Tables

**Table 1:** Chemical composition properties of bread samples per 100 g (wet basis).

Sample	a_w_	MC	Ash	Fat	Protein	Carbohydrate	TDF	EV (Kcal/100 g)
**WB**	0.92 d	31.2 g	1.2 f	3.3 e	5.5 a	58.6 a	3.3 g	286.79 a
**F10**	0.92 d	34.2 e	2.2 e	3.6 cd	5.2 b	54.7 c	3.6 e	272.41 c
**F20**	0.95 bc	35.8 c	2.6 c	3.8 b	4.7 d	53.0 d	3.8 c	265.05 e
**F30**	1.02 a	38.5 a	3.0 a	4.0 a	4.2 f	50.1 e	4.1 a	253.88 g
**O10**	0.93 cd	33.5 f	2.2 e	3.6 d	5.1 c	55.6 b	3.6 f	274.88 b
**O20**	0.94 c	35.1 d	2.6 d	3.7 c	4.6 e	53.9 c	3.7 d	267.66 d
**O30**	0.96 b	36.5 b	2.96 b	3.8 b	4.0 g	52.6 d	3.9 b	261.11 f
**SEM**	0.3	0.1	0.5	0.2	0.1	0.15	0.2	0.4

Mean values in the same column followed by different letters are significantly different (*p* < 0.05). TDF, total dietary fibre. EV, energy value. WB, wheat bread, F10, F20, and F30, fortification levels of freeze-dried banana flour. O10, O20, and O30, fortification levels of air oven-dried banana flour. SEM, Standard Error of Mean.

**Table 2:** Colour parameters of bread crust and crumb.

Crust Colour						
Sample	L*	a*	b*	Browning Index	Chroma	∆E
**WB**	66.6 a	13.1 b	37.7 a	94.5 d	41.1 a	-
**F10**	37.0 e	7.6 c	19.8 d	69.9 f	21.2 d	35.0 c
**F20**	34.4 f	6.2 cd	16.8 e	78.3 ef	17.9 e	38.9 b
**F30**	33.2 g	5.6 d	14.7 f	89.2 de	15.8 e	41.2 a
**O10**	52.3 b	15.7 a	37.9 a	110.4 c	41.0 a	14.6 f
**O20**	47.7 c	15.7 a	34.7 b	138.2 b	38.1 b	23.4 d
**O30**	43.5 d	11.4 b	29.8 c	162.1 a	31.9 c	20.5 e
**SEM**	3.8	5.1	4.6	2.6	7.1	6.3
**Crumb Colour**						
**WB**	72.5 a	4.2 f	28.6 a	53.1 f	29.2 a	-
**F10**	43.1 b	3.7 g	15.2 g	49.1 f	15.7 g	32.2 d
**F20**	41.1 d	5.2 e	18.5 f	67.4 e	19.2 f	33.0 cd
**F30**	38.6 f	7.1 c	20.1 e	85.1 d	21.4 e	35.1 b
**O10**	42.5 c	5.6 d	25.4 d	95.9 c	25.9 d	30.8 e
**O20**	39.4 e	7.7 b	27.3 b	122.1 b	28.3 b	33.3 c
**O30**	36.6 g	8.2 a	26.6 c	133.5 a	27.9 c	36.2 a
**SEM**	6.3	2.1	4.1	9.8	2.6	0.3

Mean values in the same column followed by different letters are significantly different (*p* < 0.05). WB, wheat bread, F10, F20, and F30, fortification levels of freeze-dried banana flour. O10, O20, and O30, fortification levels of air oven-dried banana flour. SEM, Standard Error of Mean.

**Table 3:** Starch digestibility of bread samples (g/100 g db).

Sample	RS (%)	SDS (%)	RDS (%)	DS (%)	TS (%)
**WB**	2.17 g	10.2 c	59.2 a	71.6 ab	71.6 a
**F10**	3.20 e	13.9 bc	55.2 b	72.7 a	72.3 a
**F20**	4.33 c	15.6 b	57.1 ab	69.1 abc	77.1 a
**F30**	8.55 a	17.8 a	50.1 d	67.9 bc	76.4 a
**O10**	2.61 f	10.4 c	54.9 bc	70.6 ab	68.1 b
**O20**	3.52 d	13.4 bc	57.1 ab	65.4 c	74.1 a
**O30**	5.92 b	15.9 b	53.9 c	69.8 ab	75.7 ab
**SEM**	0.03	1.2	1.5	1.5	1.5

Mean values in the same column followed by different letters are significantly different (*p* < 0.05). RS, resistant starch, SDS, slow digestible starch, RDS, rapidly digestible starch, DS, digestible starch, TS, total starch, WB, wheat bread, F10, F20, and F30, substitution levels of freeze-dried banana flour with wheat flour. O10, O20, and O30, substitution levels of air oven-dried banana flour with wheat flour. SEM, Standard Error of Mean.
